# Multimodal Representation Learning for Place Recognition Using Deep Hebbian Predictive Coding

**DOI:** 10.3389/frobt.2021.732023

**Published:** 2021-12-13

**Authors:** Martin J. Pearson, Shirin Dora, Oliver Struckmeier, Thomas C. Knowles, Ben Mitchinson, Kshitij Tiwari, Ville Kyrki, Sander Bohte, Cyriel M.A. Pennartz

**Affiliations:** ^1^ Bristol Robotics Laboratory, University of The West England Bristol, Bristol, United Kingdom; ^2^ Department of Computer Science, Loughborough University, Loughborough, United Kingdom; ^3^ Center for Mathematics and Informatics, Amsterdam, Netherlands; ^4^ Intelligent Robotics Group, Aalto University, Helsinki, Finland; ^5^ Department of Computer Science, University of Sheffield, Sheffield, United Kingdom; ^6^ Department of Cognitive and Systems Neuroscience, University of Amsterdam, Amsterdam, Netherlands

**Keywords:** predictive coding, multisensory integration, place recognition, sensory reconstruction, whisker tactile

## Abstract

Recognising familiar places is a competence required in many engineering applications that interact with the real world such as robot navigation. Combining information from different sensory sources promotes robustness and accuracy of place recognition. However, mismatch in data registration, dimensionality, and timing between modalities remain challenging problems in multisensory place recognition. Spurious data generated by sensor drop-out in multisensory environments is particularly problematic and often resolved through adhoc and brittle solutions. An effective approach to these problems is demonstrated by animals as they gracefully move through the world. Therefore, we take a neuro-ethological approach by adopting self-supervised representation learning based on a neuroscientific model of visual cortex known as predictive coding. We demonstrate how this parsimonious network algorithm which is trained using a local learning rule can be extended to combine visual and tactile sensory cues from a biomimetic robot as it naturally explores a visually aliased environment. The place recognition performance obtained using joint latent representations generated by the network is significantly better than contemporary representation learning techniques. Further, we see evidence of improved robustness at place recognition in face of unimodal sensor drop-out. The proposed multimodal deep predictive coding algorithm presented is also linearly extensible to accommodate more than two sensory modalities, thereby providing an intriguing example of the value of neuro-biologically plausible representation learning for multimodal navigation.

## 1 Introduction

The study of biology and the brain has inspired many innovative and robust solutions to hard problems in engineering. Biologically inspired machine learning has great potential for robotics and automation Sunderhauf et al. (2018) with significant progress being made in perception Giusti et al. (2016); Eitel et al. (2015) and scene understanding Eslami et al. (2018); Badrinarayanan et al. (2017); Gu et al. (2019); Sheppard and Rahnemoonfar (2017). Supervised deep learning takes biological inspiration from layered neural connectivity, synaptic plasticity, and distributed computation to learn non-linear mappings between inputs and desired outputs. These approaches usually rely on biologically implausible learning principles. Closer to neurobiology, deep reinforcement learning also leverages these bio-inspired architectural properties but instead learns against a task specific cost function Mnih et al. (2015). Both approaches require an error signal that is either back propagated or otherwise distributed through the layered network weight space during training. Unsupervised learning in neural networks does not typically require a globally distributed error signal for training, instead they find and exacerbate patterns in the input space by learning correlations or through local competition, typically to enable a useful reduction in dimensionality. These low dimensional latent representations of input are often used to perform clustering of complex data or serve as efficient pre-processing for a supervised or reinforcement learning back-end. All of these approaches to machine learning adopt the same assumed flow of information through the network, namely, from sensory input toward an appropriate output representation. The flow of information in a Predictive Coding Network (PCN) is both from sensory input to output and the opposite, i.e., each layer in the network predicts representations of the previous layer in parallel, ultimately predicting the actual input being passed into the lowest layer of the network Rao and Ballard (1999); Spratling (2017). Prior to learning layer-wise predictions are randomly initialised, during weight learning and inference they are compared to the predictions received from the previous layers. Local learning rules are then applied to update weights and infer neural activity in each layer to minimize the error in predictions (which is related to the free-energy or “surprise” in the system Friston (2010)) on subsequent exposures to similar input. This approach to learning is more biologically plausible as a globally distributed error is not required to update the weights, instead local Hebbian-like rules are applied Dora et al. (2018). Moreover, predictive coding is also an unsupervised learning approach and, hence, does not require labelled datasets for training. It has been used in robotics for learning sensorimotor models Park et al. (2012); Nagai (2019); Lanillos and Cheng (2018) and for goal directed planning in visuomotor tasks Hwang et al. (2017); Choi and Tani (2018). To the best of our knowledge, it has not been used for place recognition. Place recognition is the ability to interpret and recall sensory views of the world to inform an estimate of location or pose, with visual place recognition being its most common and well-studied form Lowry et al. (2016). A place recognition system is typically decomposed into two sub-systems, an image/sensory processing module, and a mapping module which stores either a metric or relative association between sensory views and the pose of an agent. In this study we primarily focus on sensory pre-processing for place recognition, our interest being in the performance of PCNs to transform samples from co-localised but disparate sensory modalities into a representation suitable for efficiently determining proximity between locations in an environment. This is interesting for two reasons, firstly, PCN is a mechanistic implementation of the parsimonious theory that perception arises from generative, inferential representations of what causes sensory inputs to arise Helmholtz (1867); Gregory (1980); Mumford (1992); Pennartz (2015); Friston (2010). This framework describes many of the phenomenological and anatomical observations from animal behaviour and neurophysiology. By incorporating this model into robots we have the opportunity to reproduce these observations in an autonomous agent and thus better understand the principles at work in the brain from an algorithmic level of abstraction. Secondly, combining information from different sensory modalities in mobile robots overcomes unimodal aliasing and sensor drop-out but introduces new challenges such as dimensionality mismatch and registration Khaleghi et al. (2013). Sensor fusion is a well-established field of research with numerous approaches developed and successfully incorporated into widespread use. The three predominant approaches of sensor fusion are probabilistic, evidential, and model-free/neural networks, with Kalman filtering Roumeliotis and Bekey (1997), Dempster-Shafer Theory Murphy (1998), and Variational Auto-Encoders (VAEs) Kingma and Welling (2013); Suzuki et al. (2017); Korthals et al. (2019) being prominent examples of the respective approaches. Auto-Encoders are well established tools in machine learning for approximating a higher dimensional input space using a lower dimensional representation space. A VAE is a generative modelling approach that uses variational inference methods for training with large-scale and high dimensional data sets Kingma and Welling (2013). More recently, this has been extended for learning bi-directional, joint distributions between different sensory modalities Suzuki et al. (2017); Korthals et al. (2019). This allows inferences in one sensory modality based on evidence in another modality *via* a jointly trained generative model. Suzuki et al. Suzuki et al. (2017) demonstrated that visual images and textual labels could be associated using a Joint Multimodal VAE (JMVAE) such that either modality could be used to reconstruct meaningful inferences about the other modality. Korthals et al. Korthals et al. (2019) used a modified version of JMVAE to jointly infer coloured geometric objects from visual and LiDAR data gathered from a simulated robot. Here we introduce a Multimodal Predictive Coding Network model (MultiPredNet) which is rooted in neurobiology and psychology, and utilise JMVAEs with the visual tactile datasets gathered in this study as a contemporary machine learning approach for comparison. The MultiPredNet presented here also fits the *model-free* learning category of sensory fusion as, similar to VAEs, it requires a period of training before it can be reliably used. Both JMVAEs and MultiPredNet learn the structural properties of the sensory information pertaining to the environment in which they are trained, i.e., they are computationally equivalent but differ in their algorithmic approach, including the learning rules. In this study we train both a contemporary JMVAE and the novel MultiPredNet using visual and tactile sensory data sets sampled from a biomimetic robot as it explores the world. The robot head has been physically designed to mimic a whiskered rat, with an array of individually actuated tactile whisker sensors and wide angle cameras in place of eyes. The dimensionality, timing and registration, or reference frame, of these two sensory modalities are different, with the salient information available from each being dependent on the current pose of the robot. Intuitively, combining cues from both sensory systems should reduce ambiguity in place recognition which ultimately will result in less frequent incorrect re-localisations from a robot pose mapping module. We extend this further by testing the ability of JMVAE and MultiPredNet to generalise between poses through the representation space itself; in other words, subsuming some of the functionality of a mapping module by the sensory preprocessing. To demonstrate the extensibility and applicability of MultiPredNet more explicitly to the robot localisation problem we use a simulation of the robot within a larger scale environment to reveal examples of loop-closure detection through its integration with a simple associative memory. Finally, we compare separate data sets that cover similar regions of the pose space to apply more conventional precision-recall curve analysis as a second measure of performance for place recognition by MultiPredNet.

## 2 Results

### 2.1 Experimental Procedure

#### 2.1.1 Data Capture

**FIGURE 1:**
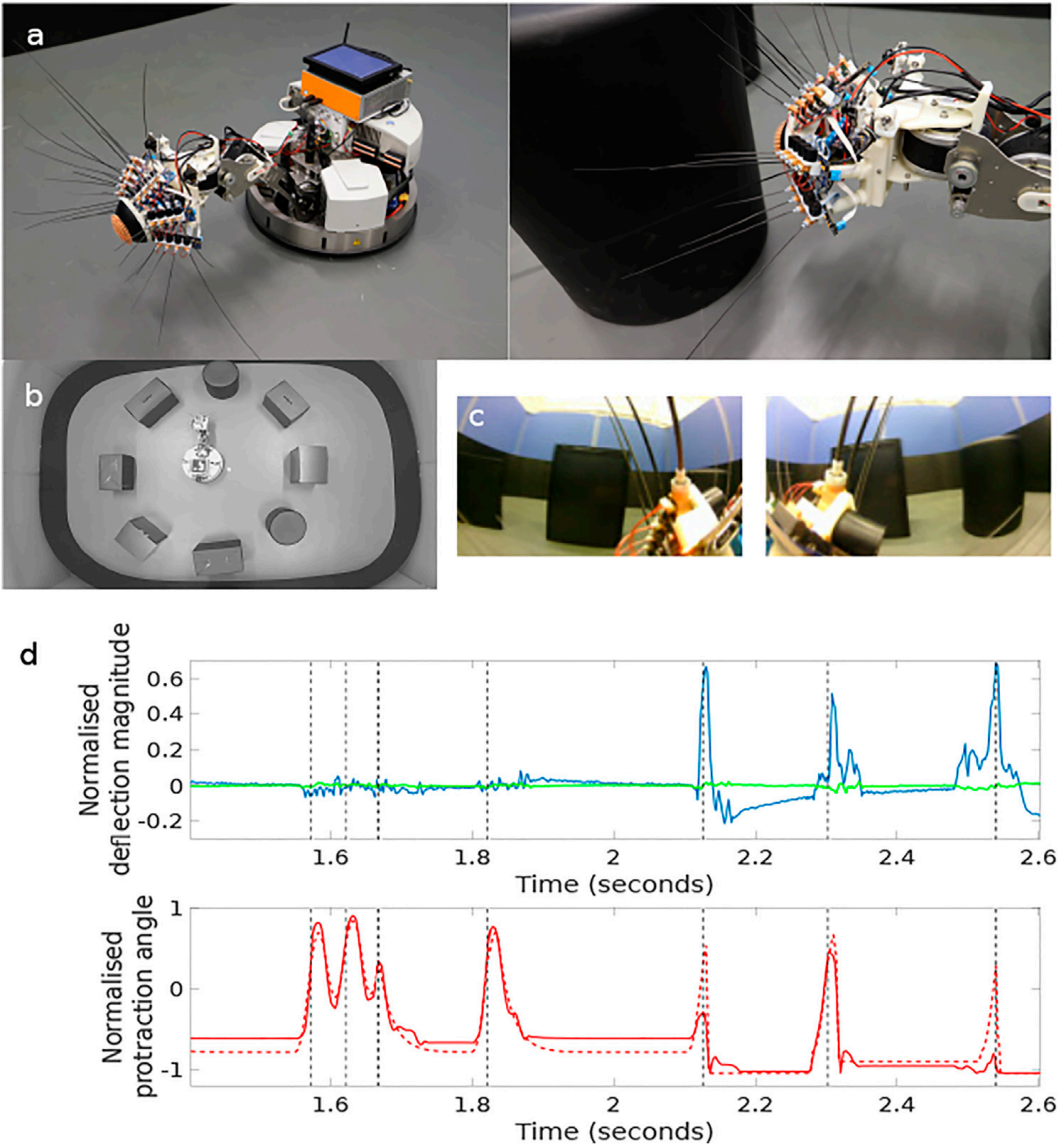
The WhiskEye robot **(A)** has 24 actuated tactile whiskers and camera eyes on its head mounted at the end a 3 dof neck and omnidrive Robotino body. Simultaneous visual frames [panel **(C)**, left and right eye cameras] and tactile samples are taken at the point of peak whisker protraction as indicated by the black vertical dashed lines in the plots of panel **(D)**. The example time series data shown in panel d are taken from a single whisker through 7 whisk cycles with only the final 3 whisks making contact with an object. The red dashed trace in the lower plot is the drive or desired protraction angle of the whisker scaled to ±1 of the full whisk angle range of ±80 degrees of rotation. The solid red line is the measured protraction angle of the whisker (*θ*_*whisk*_) which can be inhibited by contacts as is clear on the fifth whisk. The blue trace in the upper plot is the *x*- and the green the *y*-deflection of the whisker scaled to ±1 of maximum deflection magnitude. The three positive whisker contacts are clear in the final three whisks of this sample. The *x*, *y*, and *θ*_*whisk*_ samples taken at the point of peak protraction for all 24 whiskers constitute the tactile “view” of the robot at that instance. The experimental arena shown in panel **(B)**, was populated with matt black cylinders and boxes, the configuration was changed between collecting data to train the networks and for testing.

The study was conducted using a custom built robotic platform called “WhiskEye”, modelled on previous whiskered mobile robots developed in collaboration with biological scientists Prescott et al. (2009); Pearson et al. (2013). The body of WhiskEye is a Robotino™ mobile platform from Festo Didactic with an additional embedded computer and a 3 degree of freedom neck installed as shown in Figure 1. The head, which was mounted as the end-effector of the neck, has 24 individually actuated artificial tactile whisker sensors and two forward facing cameras (eyes). The embedded computer collected all sensory data and coordinated the motor action of the platform using the Robot Operating System (ROS) execution framework (Stanford Artificial Intelligence Laboratory et al., 2018). The actions of the robot were directed and controlled using a model of tactile attention distributed across functional models of distinct regions of the brain. In brief, the collective behaviour of this model was to direct the nose of WhiskEye toward the most salient region of a head centred map of space representing the volume surrounding WhiskEye’s head Mitchinson and Prescott (2013). WhiskEye’s whiskers, which occupy this space, can be actively rotated around their base mimicking the cyclic whisking behaviour expressed by many small mammals such as mice and rats Gao et al. (2001). If a whisker makes contact with a solid object during a whisk cycle then the sensory consequences of that collision will be interpreted as a more salient location in the head centred map, thus increasing the likelihood of the robot orienting its nose toward the point of contact. An orient is enacted when the saliency of a point in the map exceeds a certain level; this can be excited by whisker contacts or through a random background noise pattern that increases in relation to the time since a previous orient. In this way the robot moves through the world through a sequence of regular orients whilst preferentially attending to objects that it encounters with its whiskers. To prevent repetitive orienting behaviour a mechanism based on visual inhibition of return was included to temporarily suppress the salience of regions in the map that have recently been explored. In addition, there was a low level reflexive behaviour built into the whisker motion controllers such that the drive force to each whisker was inhibited by deflection of the shaft. This reduces the likelihood of damage and constrains the magnitude of deflections measured by the whisker, effectively normalising the sensor range Pearson et al. (2013). The data sets that were collected for this study were composed of samples taken from the whisker array and the forward facing cameras at the point of peak protraction of the whisker array. Alongside these data were stored the robot odometry, motor commands, and ground truth 2D location and orientation of WhiskEye’s head as determined by an overhead camera and associated robot mounted markers. For longer duration and larger scale experiments a simulation of the WhiskEye platform was instantiated into an on-line robotics simulator called the NeuroRobotics Platform (NRP) Falotico et al. (2017). The interface with the NRP is based on ROS which enabled the simulated WhiskEye robot to use the same control software and capture the same format of sensory data as the physical platform. Gaussian noise was added to the simulated tactile sensory responses to match statistics from the physical whiskers and the resolution of the visual frames captured from the simulated cameras were scaled appropriately to match.

**FIGURE 2:**
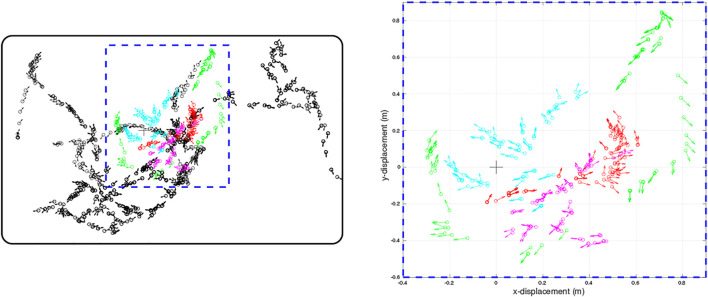
Quiver plots of the poses of WhiskEye’s head (defined as x,y position and head direction) at each sample point taken for the training set (black) and for each of the test sets (red, cyan, magenta, green) used to evaluate the models. Each test set was recorded from a different initial pose of WhiskEye and in the arena populated with different object configurations to test for generalisation. The right panel is a scaled view of the region indicated by the blue dashed rectangle in the left panel. The bold black rectangle enclosing the sample points in the left panel indicates the boundary walls of the arena. The arena measured 3.5 m by 2.25 m

**FIGURE 3:**
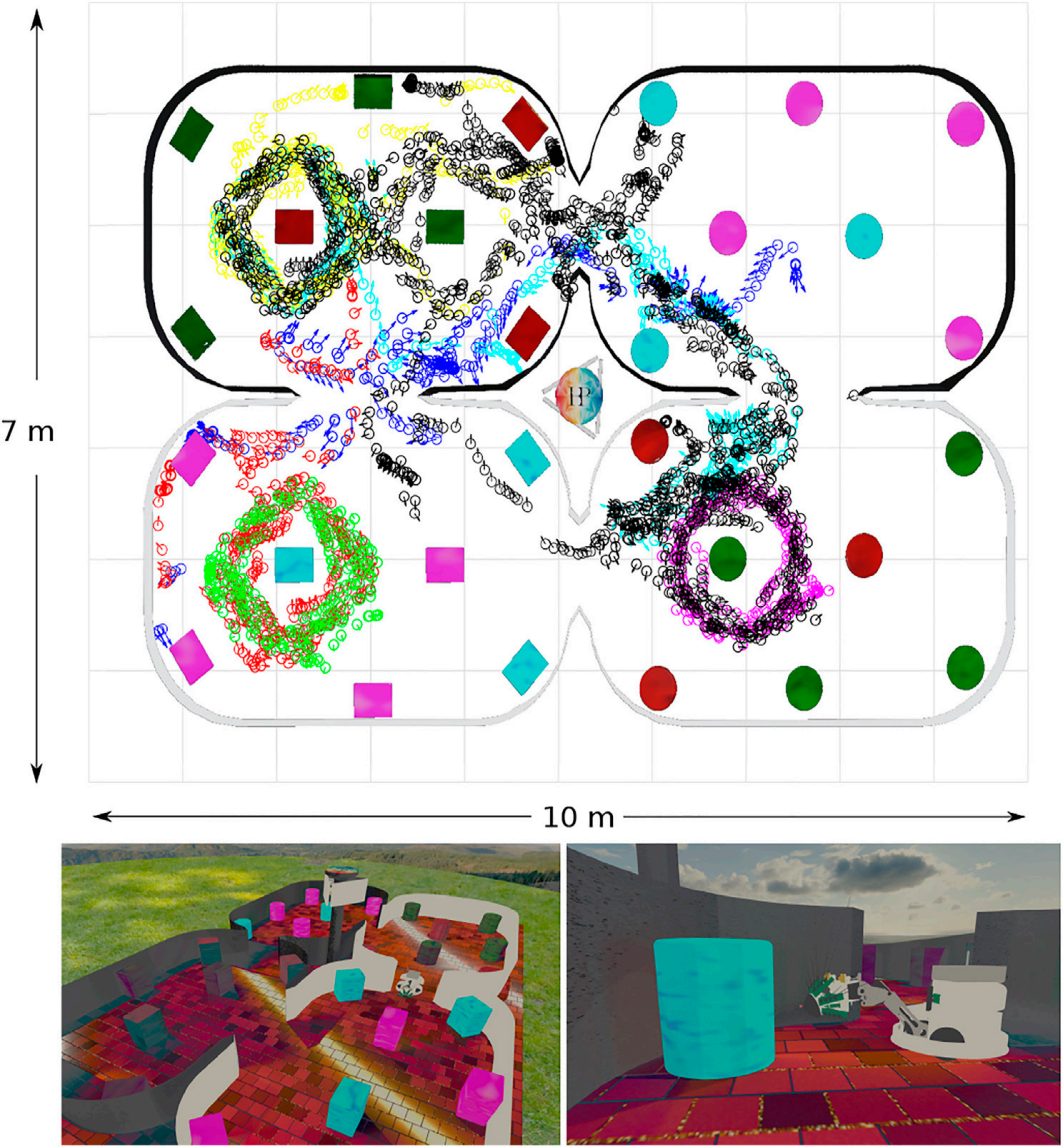
Simulated WhiskEye in the NeuroRobotics Platform. Top panel) Quiver plot of head poses (*x*, *y*, *θ*) at sample points taken as the training set (black) and 6 test sets (blue:1, red:2, green:3, magenta:4, cyan:5, yellow:6) The arena walls and coloured objects have been superimposed onto the quiver plot for reference. Lower panels) Screen shots taken from the simulator showing the arena and simulated WhiskEye robot as it explores the arena. The tactile attention model used to control the physical platform is the same as used in the simulator.

The physical environment was bounded by a 600 mm high ellipsoid shaped perimeter measuring 3 m by 5 m which in turn was bounded by 1.5 m high blue partition boards on a smooth grey painted concrete floor to minimise distinct visual cues. Within the bounded arena we placed black coloured 600 mm high boxes and cylinders in various configurations to delineate different environments for gathering training and test data sets. The training set was gathered in batches as the robot explored the training arena which was then concatenated together into 1,270 visual-tactile data points representing 30 min of real time exploration (nominal whisk rate 1 Hz). Test sets were gathered from arenas composed of differently configured geometric shapes (Figure 1B) in batches of 73 samples for each set. The trajectory of the robot was governed by the attention driven model of control and therefore not repeatable between test runs. However, the robot did adopt similar poses at multiple points between the test data sets and training set as shown in the quiver plots of Figure 2. The simulated environment consisted of four interconnected quadrants each the same size as the physical arena but with alternate black and white walls and different coloured and configured cylinders and boxes in each quadrant. A training set of 2,400 visual-tactile samples and 6 test sets of 400 samples each were captured as the simulated robot explored in different regions of the environment (see Figure 3). This simulated arena serves as a controlled intermediate step toward larger scale unstructured environments to systematically test the efficacy of MultiPredNet for robot localisation. Specifically, we used the simulator here to perform longer duration experiments in order to capture loop closure events between data sets as is clear in the quiver plots of Figure 3.

#### 2.1.2 Comparative Network Model Architecture

**FIGURE 4:**
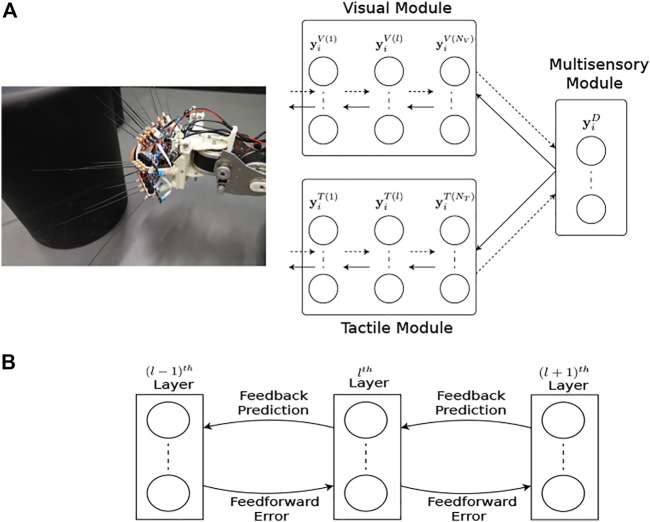
Multimodal predictive coding network architecture. **(A)** The network consists of 3 neural network modules; the Visual and Tactile modules taking visual and tactile input from the WhiskEye robot with *N*_*V*_ and *N*_*T*_ layers respectively; and the Multisensory module consisting of a single layer that predicts the activities of the neurons in the last layers of both the visual and tactile modules. Note that each layer in the Visual and Tactile modules are also predicting the activities of neurons in their preceding layers as shown in panel **(B)**, passing prediction errors forward through the network.

The network structure and learning rules for the proposed MultiPredNet and the VAE are presented in the materials and methods section. Briefly, the MultiPredNet consists of 3 modules such that one module (called the visual module) receives visual data as input, the second module (called the tactile module) receives tactile data, and the third module (called multisensory module) receives the concatenated higher order representations inferred by the visual and tactile modules (see Figure 4). The synaptic weights of the three modules are learned using the same Hebbian-like learning rule. The representation inferred from the last layer of the multisensory module denotes the joint representation inferred using MultiPredNet. We compared the place recognition performance of MultiPredNet with existing VAE approaches for inferring multisensory representations, namely Joint Multimodal VAEs (JMVAEs) or more specifically a JMVAE-zero and JMVAE-kl (Suzuki et al., 2017) as shown in Figure 14. JMVAE-zero consists of two VAEs for handling visual and tactile inputs respectively. The last layer of the encoders in both VAEs is connected to a common layer whose activities are used for multisensory place recognition. JMVAE-kl uses the same network architecture as used in JMVAE-zero with two additional VAE encoders that infer unisensory representations based on visual and tactile inputs respectively. The multisensory and unisensory representations in JMVAE-kl are optimised together to be similar to each other using a Kullback-Leibler divergence component in the objective function. This allows JMVAE-kl to generate better crossmodal reconstruction in case of sensor drop-out. For a fair comparison, the dimensions of the multisensory representations obtained from MultiPredNet, JMVAE-zero and JMVAE-kl were fixed at 100.

#### 2.1.3 Performance Metrics

To quantitatively measure the performance of a place recognition system we need to relate the ground truth 3D pose of the robot head [(*x*, *y*, *θ*_*head*_) relative to a global reference frame] to the representations generated by the sensory pre-processing modules. It leads, therefore, that to perform efficient place recognition the similarity between representations should correlate with similarity between robot poses. Here we adopt a technique from computational neuroscience called Representational Similarity Analysis (RSA) that was originally developed to compare measurements from brain activity, behavioural measurements and computational modelling Kriegeskorte et al. (2008). For the current study, we computed a Representational Dissimilarity Matrix (RDM) for both pose and the generated representations from candidate systems for each run of the robot in the testing arena and compared their rank order using Spearman’s rank correlation. Briefly, an RDM is a symmetric matrix around a diagonal of zeros with each element encoding the dis-similarity of the row sample to the column sample, i.e., the distance from each sample in the data set to all the others. Comparing the rank order of the RDMs for ground truth pose against representations provides an intuitive measure of performance for place recognition. A second measure of performance in this study was the error in inferring the tactile (visual) modality based on the representations inferred from the MultiPredNet and JMVAEs in presence of visual (tactile) modality (i.e., by sensor drop-out). This experiment was carried out using the physical test data only. To evaluate the extensibility of MultiPredNet and the validity of RSA as a measure of performance for place recognition in larger scale, on-field settings we use the simulated data sets and a simple mapping system to reveal loop closure recognition as a qualitative demonstration of its performance in robot localisation. This is evaluated further using more conventional ROC curve analysis between 2 of the simulated testsets that have similar but not identical ground truth trajectories.

### 2.2 Model Performance at Place Recognition

**FIGURE 5:**
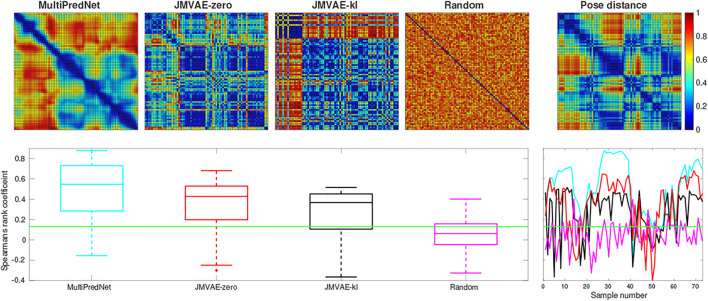
Representational Similarity Analysis of three trained network models to measure performance at place recognition across a small example test set. The top row presents the Representational Dissimilarity Matrix (RDM) generated from **(left to right)** MultiPredNet, JMVAE-zero, JMVAE-kl and random representations in response to visual and tactile samples taken from physical test set 1 (73 samples). The RDM on the far right was generated from the associated ground truth 3D poses of WhiskEye’s head (*x*, *y*, *θ*_*head*_) for each sample in the test set. The boxplots in the lower panel summarise statistics of the Spearman’s rank correlation coefficient (*ρ*) calculated between the pose and representation distances across the test set for each model as shown in the coloured line plots in the panel to the right (cyan: MultiPredNet, red: JMVAE-zero, black: JMAVE-kl, and magenta: Random). The Green horizontal line indicating 99% significance above chance (*p* < 0.001, *N* = 73).

The three models, namely MultiPredNet, JMVAE-zero and JMVAE-kl, were trained on the same physical data set which was shuffled and decomposed into mini-batches of 10 samples. Each model was trained for 200 epochs. Once trained, the physical test sets were presented to each model one sample at a time to infer corresponding sets of joint latent representations. These were used to estimate RDMs for corresponding models which were used to compute the rank order with respect to the RDM of corresponding poses of the robot from each of the four test sets (see Figure 2 for ground truth poses). Figure 5 contains example RDMs displayed as heatmaps from typical instances of each model validated against test set 1. The boxplots summarise the statistics of the Spearman’s rank correlation coefficient (*ρ*) calculated for each sample in the test set, with (*p* < 0.001, *N* = 73) indicated by the horizontal green line. For control, a random set of representations was also compared to reveal the structural relationship that each model has found between pose and multisensory view.

Using the same analysis across all test data sets, *N* = 292, the average *ρ* and the percentage of samples that scored above statistical significance (*p* < 0.001, *N* = 292) were (0.289, 69.17*%*) for MultiPredNet, (0.141, 47.26*%*) for JMVAE-zero, and (0.140, 49.31*%*) for JMVAE-kl. Applied to place recognition, a true positive correlation between representation distance and pose distance will result in a correct re-localisation. Therefore, we can expect the frequency of true positive re-localisations generated by the MultiPredNet to be 20−22*%* higher than JMVAE.

**FIGURE 6:**
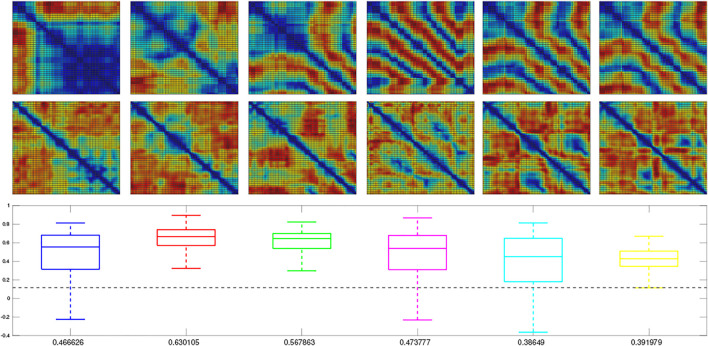
Representation Similarity Analysis applied to each of the 6 test sets sampled from the simulator and inferred by a trained MultiPredNet Model using same network topology as for physical data sets. Top row) Representation Dissimilarity Matrices (RDMs) for the first 100 samples of pose from each set (1–6 left to right). Middle row) RDMs for the first 100 inferred joint latent representations from each set. Lower panel) Box plots summarising the Spearman’s Rank coefficient (*ρ*) calculated for the full 400 samples of each test set. The colour of each box plot is the same colour as quiver plots for each test set shown in Figure 3, with the black dashed line indicating significance (*p* < 0.001, *n* = 400). The mean value of *ρ* for each set is printed beneath each box plot for clarity.

We next trained a MultiPredNet model on the simulated training set (2,400 samples) using the same network topology, batch size, learning rates and epochs as adopted for the physical data set model. For visual clarity, the RDMs for only the first 100 samples from each of the 6 simulated test sets are shown in Figure 6, whilst the box plots summarise the statistics of *ρ* for all samples in each set (*n* = 400). As in the physical tests the above significance positive correlation is clear (mean *ρ* beneath each boxplot *p* < 0.001, *n* = 400), suggesting that MultiPredNet can infer structural relationships in the simulated sensory modalities appropriate for place recognition as in the physical demonstration.

### 2.3 Model Performance During Sensor Drop-Out

**FIGURE 7:**
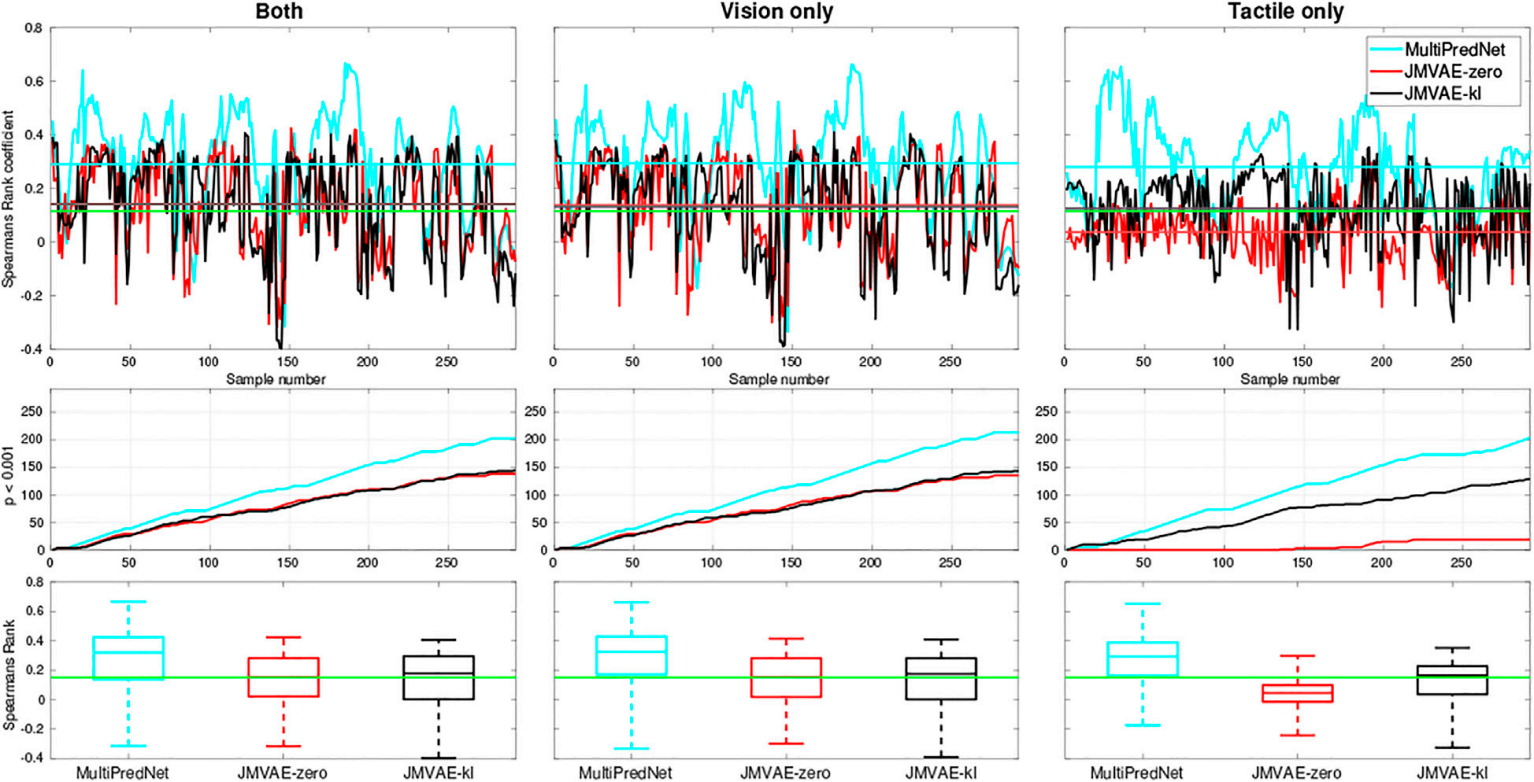
Spearman’s Rank correlation coefficient (*ρ*) to measure performance at place recognition by 3 trained models across all four physical test sets when both sensory modalities are available **(left column)**, only vision available **(middle column)** and only tactile available **(right column)**. The top row of panels trace *ρ* with the coloured horizontal lines indicating the mean value for each model and green line indicating significance (*p* < 0.001, *N* = 292). The middle row of traces show the cumulative number of samples across the test set that scored an above chance positive correlation in each of the sensory conditions. The statistics for *ρ* across each model is distilled into boxplots in the bottom row, again with the green line indicating significance.

The models were also evaluated for place recognition in a sensor drop-out scenario. For this purpose, we evaluated the place recognition performance of the three models using either visual or tactile input with the other sensory modality set to zeros. All three models performed well at place recognition (average *p* < 0.001) across each physical test set when only visual sensory information was available (see Figure 7). However, with only tactile sensory information available the JMVAE-zero model could not maintain the positive correlation between representations and ground truth pose data above the significance threshold in the majority of cases. These results are presented in Figure 7, which summarises the Spearman’s rank correlations for each model across all four test sets with both sensory modes available, only vision, and only tactile available. The line plots in the bottom panels track the cumulative number of samples that returned an above chance positive correlation (*p* < 0.001) between pose and representation distance implying a positive contribution towards place recognition. In summary, the mean *ρ* and *p* < 0.001 percentage scores for each model in the two drop-out conditions, only vision available and only tactile available, were (0.294, 72.95*%*) and (0.279, 69.52*%*) for MultiPredNet, (0.138, 46.23*%*) and (0.036, 6.51*%*) for JMVAE-zero, and (0.131, 48.97*%*) and (0.126, 44.18*%*) for JMVAE-kl. These results indicate that the MultiPredNet model has the potential to correctly re-localise on average 25*%* more often than both the JMVAE based models in the absence of either tactile or visual cues (*p* < 0.001).

As these models are generative in nature we can compare their ability to reconstruct the missing modality inferred from the conditioned evidence derived from the other. Indeed, the loss function used during training of the three models is computed using the reconstruction error. During training the JMVAE models generate sensory reconstructions by propagating the joint latent representation through a decoder network which is trained end-to-end with the encoder network using back-propagation. In contrast, all layers of the MultiPredNet model generate predictions about the activity of neurons in the previous layer of the network and the error in these predictions is used to update the weights during training according to a Hebbian-like learning rule Dora et al. (2018). In the absence of input in a given sensory modality, the joint latent representation inferred using a single modality is propagated backwards, by way of feedback projections to the input layer, to reconstruct the sensory input in the missing modality.

**FIGURE 8:**
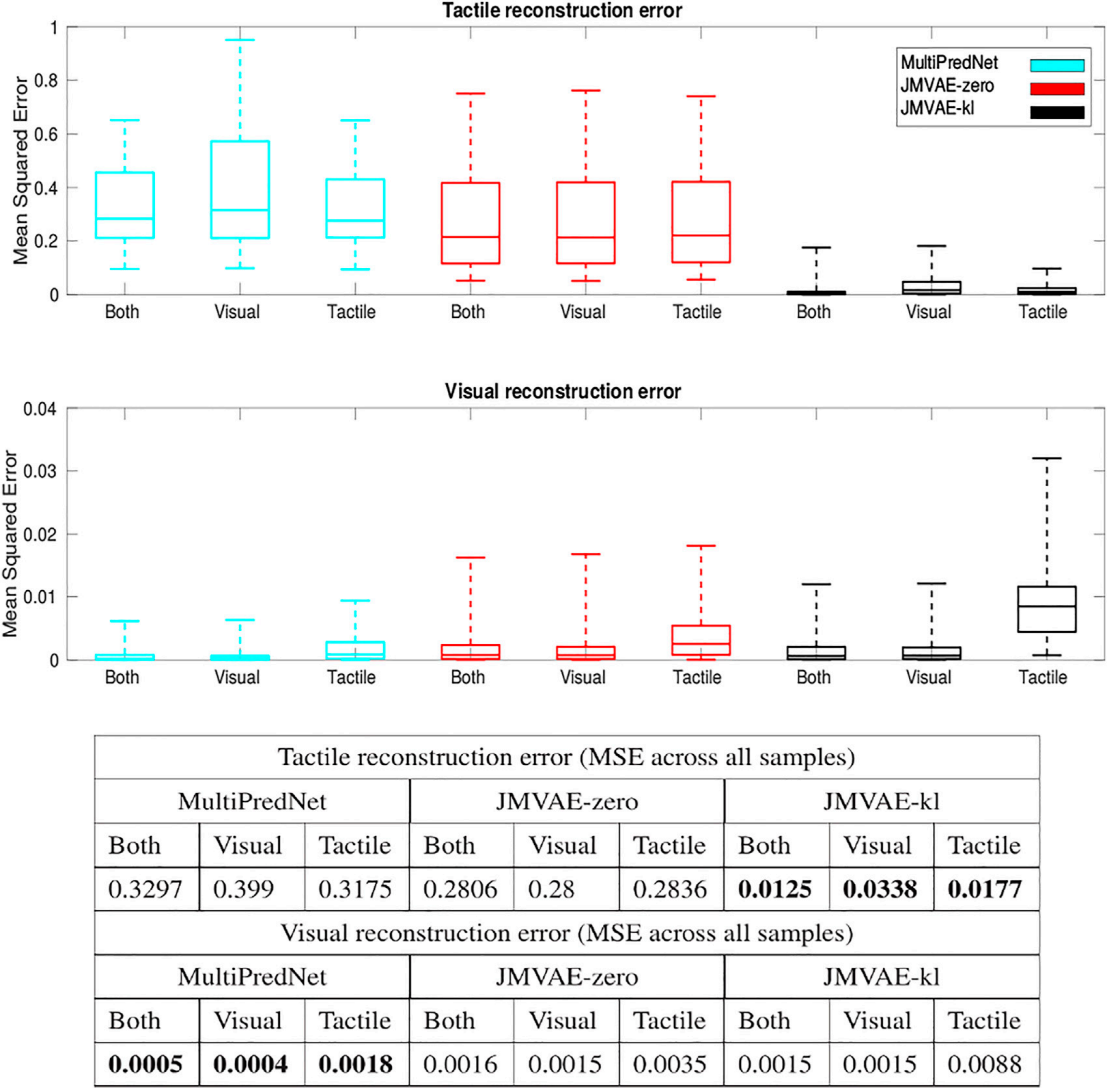
Sensory reconstruction errors from the 3 trained model networks in response to all four concatenated test sets under 3 test conditions; *Both* visual and tactile sensory data available; *Visual* data available and tactile masked; and *Tactile* data available with visual data masked. The top panel box plots summarise statistics of the mean squared error between actual tactile sensory data and the reconstructed tactile impressions generated by each of the networks. The middle panel applied the same analysis to the visual reconstructions with the average MSE for each model in each of the conditions summarised in the table beneath (bold highlighting lowest error condition).

**FIGURE 9:**
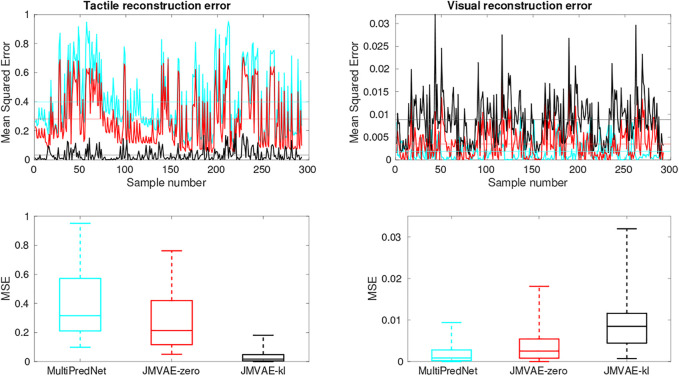
Tactile and Visual reconstruction errors from each sample in all test sets inferred by the MultiPredNet, JMVAE-zero and JMVAE-kl models with the tactile (and visual respectively) sensory input obscured. Note the systematic offset in tactile reconstruction error from the MultiPredNet and JMVAE-zero model as indicated by the mean of the Mean Squared Error (MSE) across all samples (horizontal coloured lines).

The Mean Squared Errors (MSE) for the tactile and visual reconstructions from each of the network models in the three sensory conditions are plotted in Figure 8. The results from the JMVAE-kl model revealed that it had successfully accommodated a systematic positive off-set in the tactile reconstruction which both the JMVAE-zero and MultiPredNet had failed to (See Figure 9). With this off-set removed, the tactile reconstruction errors from the JMVAE-kl model were still significantly lower than the other two (*p* < 0.001). Another interesting observation was that the JMVAE-kl model performed worse (relative to the others) at visual reconstruction when only tactile sensory input was available. However, as with the MultiPredNet, it performed consistently at place recognition under this condition on which JMVAE-zero failed to maintain as shown in Figure 7. This suggests that performance on place recognition (measured by *ρ*) and sensory reconstruction (as measured by MSE) are not correlated.

### 2.4 MultiPredNet Performance at Robot Localisation in Simulated Field Trials

**FIGURE 10:**
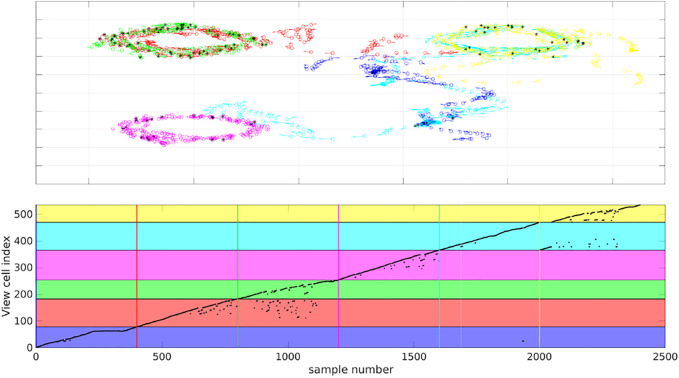
View-cell memory sequentially presented with representations generated from all 6 simulated test sets to associate poses with proximal representations. Top panel) Colour coded quiver plots of ground truth poses of the simulated WhiskEye head during each of the 6 test sets (1 = blue, 2 = red, 3 = green, 4 = magenta, 5 = cyan and 6 = yellow). The black asterisks indicate samples which triggered a re-localisation event in the view-cell memory, i.e., the representation of that sample was close to a previous representation stored in the view-cell memory. Lower panel) Graph of view-cell index against sample number from the concatenated 6 test sets (*n* = 2,400). The coloured horizontal bars highlight the region within the view-cell memory that store representations encountered during a particular test set (colour matched to test sets). The vertical coloured lines set the start of each new region in the view-cell memory. Re-localisation events are marked by a step decrease in view cell index between samples, significantly, samples from test set 3 are triggering re-localisation events that reference to view-cells created in test set 2.

To evaluate place recognition by MultiPredNet more explicitly, a simple memory module based on the view-cell memory of RatSLAM Milford et al. (2004) was adopted to associate poses with the joint modal representations inferred by the MultiPredNet at each sample step. The distance (1 − Pearson correlation) between the current representation and others already stored in the memory was calculated, if this was above a certain threshold (discussed below) then it is considered novel and a new view-cell is added to the memory containing the representation and associated pose. If the distance was below the threshold, i.e., they were deemed similar, then a re-localisation event was registered and the representation not stored into memory. All 6 simulated test sets were concatenated together into a continuous run of 2,400 samples and presented to the view-cell memory in sequence. The results shown in Figure 10 demonstrate that similar poses are recalled from the view-cell memory triggered by similarity in representation. The black asterisks in the quiver plot highlight the sample points at which re-localisation events were detected by the view-cell memory, note that these occur during loop-closures within and between test sets. This is more clearly shown in the plot of the lower panel of Figure 10 where the horizontal coloured panes indicate the regions of the view-cell memory that are composed of view-cells created during each test set in sequence (blue, red, green, magenta, cyan and yellow) with the coloured vertical lines marking the start of each region. The black dots indicate the view-cell index address associated with each sample in the concatenated data set, re-localisation events, therefore, are indicated by a sharp decrease in view-cell index between samples. This is most evident during sets 2 and 3 (red and green) which include re-localisation events occurring during set 3 that reference view-cells created during set 2. Referring back to the quiver plot we can see that these relate to the loop-closures that occur in the shared pose space adopted by the robot during the acquisition of these 2 test sets. The same phenomenon is seen between sets 5 and 6 (cyan and yellow) and, to a lesser extent, between sets 5 and 1 (cyan and blue). Test set 4 (magenta) has multiple re-localisation events, however, these are confined to its own region of the view-cell memory which corresponds to the unique region of pose space that it represents.

**FIGURE 11:**
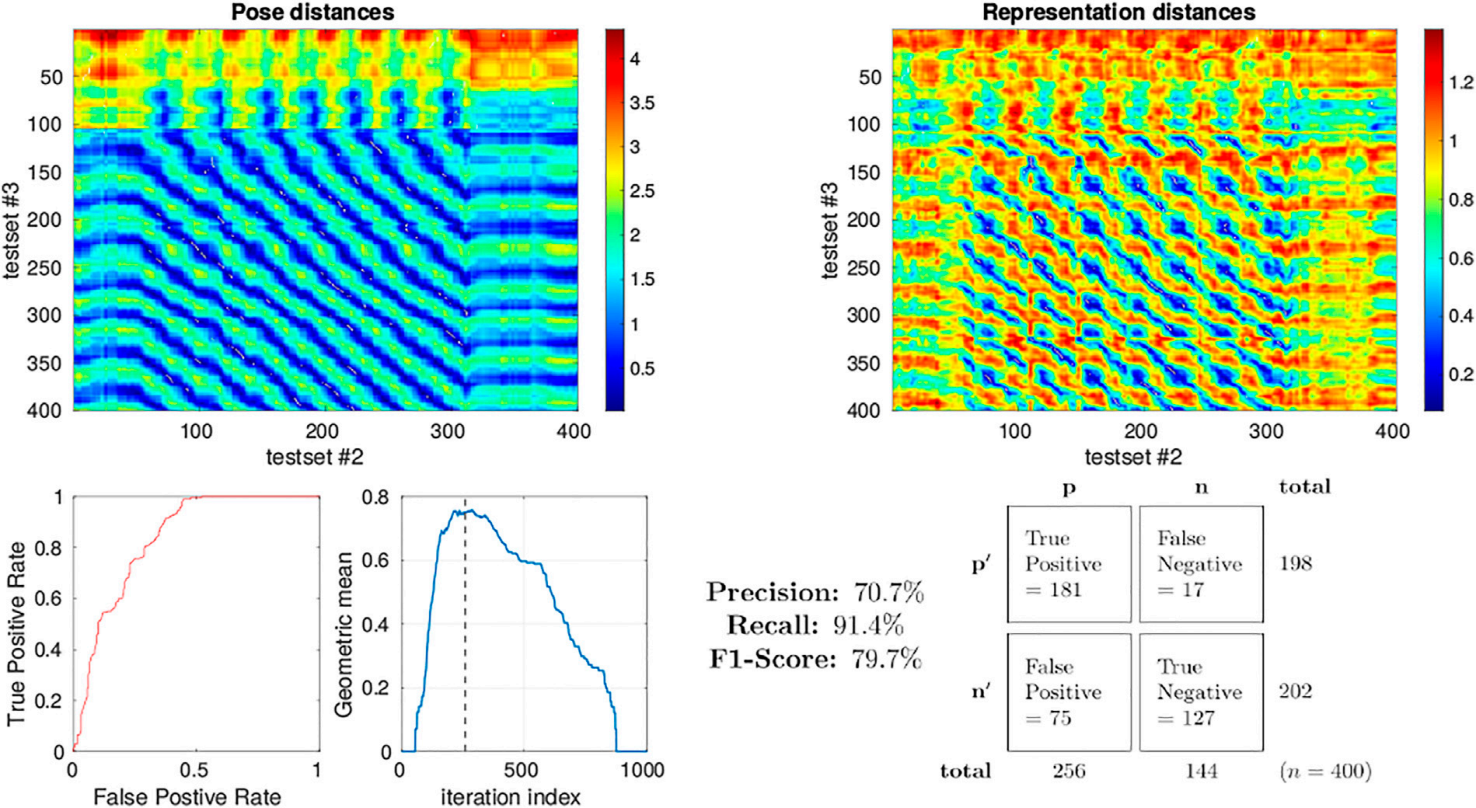
Precision-recall curve analysis applied to simulated test sets 2 and 3 (see Figure 3) classifying for place recognition through the representation space of the trained MultiPredNet. Top left) Heatmap summarising the distance in pose space from each sample in test set 2 against each sample in test set 3. Top right) Heatmap of distances in representation space between samples in test set 2 against all samples in test set 3. White dots in the heatmap indicate classification points for each sample (*n* = 400) selected as the lowest distance in representation space between the 2 test sets. These points have been translated into the pose distance heatmap to determine a true or false classification. Lower left plot) Receiver Operating Characteristic (ROC) curve summarising impact of representation threshold for determining positive versus negative classifications. Lower right plot) Geometric mean of the ROC curve at each threshold iteration with the peak highlighted by the vertical dashed line. The confusion matrix contains the summed classification classes when using the optimal representation threshold determined from ROC curve with the Precision, Recall and F1-Score for the classifier calculated from them.

A more quantitative measure for the performance of a system at place recognition can be obtained through the analysis of the precision-recall rate Kazmi and Mertsching (2016), Flach and Kull (2015). To calculate this we selected test sets 2 and 3 from the simulator as we have seen that they approximately share the pose space within the arena during their independent runs with loop-closure events evident from the qualitative analysis described above. The distance between representation of each sample in each test set to each sample in the other was calculated again using 1 − Pearson correlation as the metric of distance. The distance in pose between each sample in a set against the pose of all samples in the other set was also calculated (Euclidean). These inter test set distance matrices are displayed as heatmaps in Figure 11 allowing us to visualise which regions of both the pose and representation space are similar between the 2 test sets. Intuitively, regions of low distance in representation space should correspond to an equally low distance in pose space to perform place recognition. Putting this into the context of the view-cell memory demonstration, a below threshold representation distance should trigger a re-localisation event from one test set to the other which should correspond to a similar pose. Therefore, for each sample in test set 2 (columns in heatmaps of Figure 11) we select the sample from test set 3 (row) with the lowest distance in representation space as the candidate classification. If the distance of a classification is below a representation threshold we label it as *Positive*, if higher then it is *Negative*. The [column, row] coordinates of the candidate classifications are relayed to the pose space distance matrix to determine whether they were *True* or *False* classifications by comparing the pose distance to a threshold which we fixed arbitrarily at 0.2. To determine an appropriate representation threshold to maximise performance from this system we calculated the peak geometric mean of the Receiver Operating Characteristic (ROC) curve generated through a sweep of 1,000 threshold values from 0.001 to 1 registering the classifications generated from each as *True Positive* (TP), *False Positive* (FP), *False Negative* (FN) or *True Negative* (TN) accordingly. The area under the ROC curve was found to be 0.836 with a peak geometric mean found at iteration 290 indicating an optimal representation threshold of 0.29 to maximise the classification performance of the system. With this threshold the Precision (70.7*%*), Recall (91.4*%*) and F1-Score (79.7*%*) for the classifier was calculated.

## 3 Discussion

**FIGURE 12:**
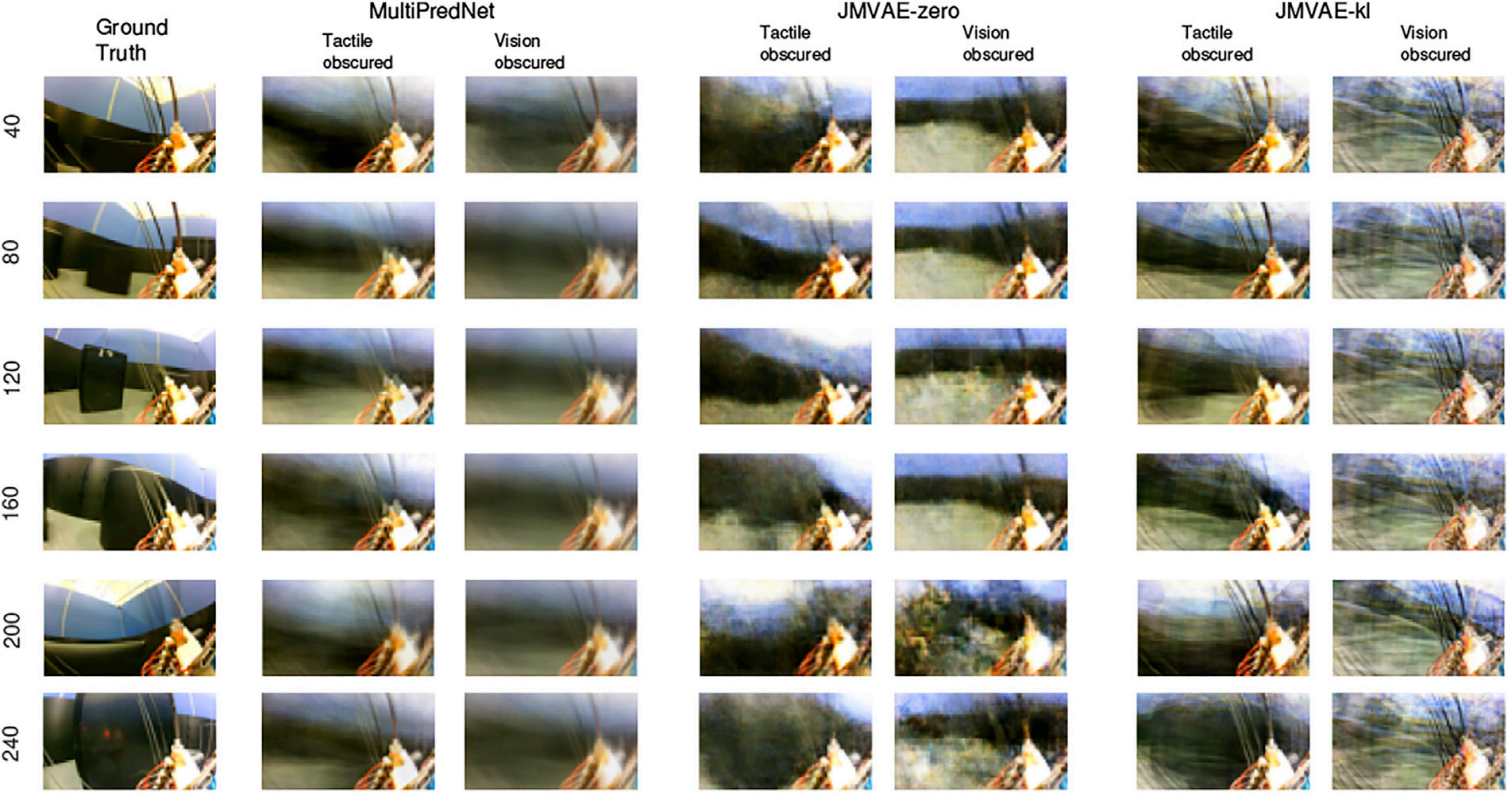
Example frames captured by the eye camera that are presented as visual input to the trained models with their subsequent reconstructions in the 2 sensory drop-out conditions. The Ground truth images in the left column were taken at the sample number indicated to the left of each panel with each reconstruction from the models presented in that row. The reconstructions are qualitatively similar in their quality across all 3 models, however, MultiPredNet did return the lowest mean squared reconstruction errors in all conditions as shown in Figure 8.

**FIGURE 13:**
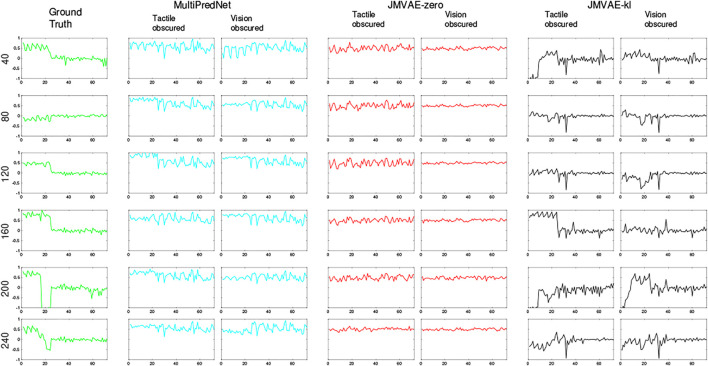
Example tactile information captured from the whisker array at point of peak protraction at the sample points in the test set as indicated by the number on the left of each row. The first 24 values in each sample is the protraction angle of each whisker (*θ*_*whisk*_) in the array with the remaining 48 values indicating the magnitude of deflection experienced by each whisker in the *x* and *y* dimensions (refer to Figure 1 for description). As in Figure 12, the plots in each row to the right of the ground truth were reconstructed by the different trained models under test during the 2 drop-out conditions. The JMVAE-kl model performed best at tactile reconstruction in all conditions (see Figure 8) as is clear from these indicative examples. Both MultiPredNet and JMVAE-zero failed to accommodate the systematic off-set in deflection angle which JMVAE-kl has, i.e., nominally zero in the last 48 values of reconstructed vector.

The potential for networks trained using predictive coding and Variational Auto Encoders for learning joint latent representations of multimodal real-world sensory scenes to perform place recognition has been demonstrated. The MultiPredNet model proposed here consistently outperformed the JMVAE-zero and JMVAE-kl models in place recognition as evident from the RSA in all 3 test conditions using the physical platform (Figure 5). Importantly, each model was composed of the same number of layers and nodes, trained and tested using the same data sets, and their weight spaces learnt through the same number of training epochs. The analysis used to compare performance at place recognition between models serves as a proxy to more direct measures of performance at place recognition through navigation. To clarify this we have demonstrated that coupling the MultiPredNet to a simple associative memory system and capturing longer duration data sets, enables more conventional metrics for quantifying place recognition to be derived. What now remains to be demonstrated is how these metrics compare to other model-free or model-based place recognition systems that combine visual and tactile sensory data. Toward this we are unaware of any suitable model for comparison other than the ViTa-SLAM system Struckmeier et al. (2019) introduced by co-authors in a previous study that inspired this work and as such would be uninformative to compare against. What we have shown is that RSA does enable an empirical comparison of complex representation spaces to low dimensional pose spaces in an intuitive manner to guide in the evaluation of candidate models and to adjust network parameters prior to full integration with a SLAM back-end. The MultiPredNet returned the lowest visual sensory reconstruction errors whilst the JMVAE-kl model performed best at tactile reconstruction (Figures 8, 12, 13). The Kullback Leibler divergence term included in the JMVAE-kl loss function during training was introduced to bring the representation spaces of the disparate sensory modalities closer to enable bi-directional multimodal reconstruction. This appears to be the case for tactile reconstruction from visual input, however, it did not result in an improved performance in place recognition nor did it improve visual reconstruction from tactile as evidenced in the lower panel of Figure 8 and example reconstructions shown in Figure 12. The large offset apparent in the tactile reconstruction errors from the JMVAE-zero and MultiPredNet models suggest that these models did not accommodate this disparity. However, the MultiPredNet model maintained an above significance correlation in place recognition when only tactile information was available, which JMVAE-zero could not. Interpreting the representation space of MultiPredNet is, therefore, subject to further investigation, which reinforces the position that VAEs are certainly better understood machine learning tools and as such are the obvious choice for adoption by robotics engineers. However, PCNs stand as an algorithmic level solution to learning that more closely approximates the physiology of a “cortical compute unit”. The base compute unit in a PCN is the same throughout the network, referred to by Rao and Ballard as a Predictive Estimator Rao and Ballard (1999), wherein only local computation and updates are performed during training and inference. By contrast the JMVAE approaches require separate decoder networks for training, which are then disregarded during inference if sensory reconstructions are not required. In the case of the JMVAE-kl network which learns unimodal representations in parallel to, and in support of, the joint modal distribution during training, the additional encoder-decoder network pairs are also disregarded during inference. In a purely software-based system this inefficiency is not an issue, however, as we look toward the future of embedded machine learning, particularly within robotics and edge based applications, we anticipate the increasing adoption of energy efficient hardware platforms, such as neuromorphic devices Krichmar et al. (2019). The immediate practical advantage in adopting the PCN approach, therefore, is that the algorithm is highly amenable to hardware optimisation through parallel distributed learning and processing. Unlike VAE networks, the local learning rule applied at each layer of a PCN requires no global back propagation of error in agreement with the physiology of mammalian cortex Roelfsema and Holtmaat (2018). Moreover, the basic feedforward-feedback structure of PCNs resemble the core architecture of the sensory neocortex Felleman and Van Essen (1991); Bastos et al. (2012); Pennartz et al. (2019). Further, the modular nature of the PCN compute unit that encapsulates the encoder-decoder pairing of the VAE but at a local level, allows a graceful scaling of the algorithm through simple duplication of the basic unit. This principle extends to including additional sensory modalities for joint representation learning, whereby any additional modality specific networks could be integrated into the multisensory network with a linear increase in complexity. By contrast, the JMVAE-kl approach would require a combinatorial increase in encoder-decoder pairs to correctly integrate additional modalities into the joint space. In conclusion, PCNs not only offer computational advantages to autonomously learning robots in terms of place recognition, but also convey a considerable neurobiological plausibility and better scalability as compared to VAE approaches.

## 4 Materials and Methods

### 4.1 Multi-Modal Predictive Coding Network Algorithm

#### 4.1.1 Multimodal Predictive Coding Network Architecture

The network consists of three modules, namely the visual module, tactile module and multisensory module as shown in Figure 4A. The visual module processes visual information and consists of a neural network with *N*_*V*_ layers. Activity of the neurons in the *l*th layer for the *i*th input is denoted by a *n*_*V*(*l*)_ dimensional vector, $y_{i}^{V\left(l\right)}$ where *n*_*V*(*l*)_ denotes the number of neurons in the *l*th layer of the visual module. Each layer in the network predicts the activity $\left(\hat{y}\right)$ of the preceding layer according to

$${\hat{y}}_{i}^{V\left(l-1\right)}=\phi {\left({\left(y_{i}^{V\left(l\right)}\right)}^{T}W_{l\left(l-1\right)}^{V}\right)}^{T}$$ (1)

where $W_{l\left(l-1\right)}^{V}$ denotes the synaptic weights of the projections between the *l* ^th^ and (*l* − 1)^*th*^ layer in the visual module and *ϕ* is the activation function of the neurons (ReLU). The lowest layer in the network predicts the visual input $\left(X_{i}^{V}\right)$ and other layers predict the activities of neurons in the preceding layer. All layers in the network generate these predictions in parallel using Eq. 1. This aspect of the network is different from commonly employed feedforward networks in deep learning, where information is sequentially propagated from the first to last layer of the network. The tactile module consists of a similar neural network with *N*_*T*_ layers that processes tactile information. The multisensory module consists of a single layer which predicts the activities of neurons in the last layers of both the visual and tactile modules. The activity patterns of neurons in this layer are denoted by $y_{i}^{D}$ for the *i*th input and serve as the representations used for place recognition.

#### 4.1.2 Learning Algorithm

Predictive coding is used to update the synaptic weights and infer neuronal activities in the network. A graphical depiction of the inter-layer connectivity is shown in Figure 4B. The *l*th layer in the visual module generates a prediction about the neuronal activities in the (*l* − 1)^*th*^ layer and also receives a prediction of its own neuronal activity from the (*l* + 1)^*th*^ layer. The goal of the learning algorithm is to infer *l*th layer neuronal activity $\left(y_{i}^{V\left(l\right)}\right)$ for the *i*th input that generates better predictions about neuronal activity in the (*l* − 1)^*th*^ layer and is predictable by the (*l* + 1)^*th*^ layer. For this purpose, $y_{i}^{V\left(l\right)}$ is updated by performing gradient descent on the error function

$$e_{i}^{V\left(l\right)}={\left({\hat{y}}_{i}^{V\left(l-1\right)}-y_{i}^{V\left(l-1\right)}\right)}^{2}+{\left({\hat{y}}_{i}^{V\left(l\right)}-y_{i}^{V\left(l\right)}\right)}^{2}$$ (2)

which results in the following update rule for $y_{i}^{V\left(l\right)}$

$$\Delta y_{i}^{V\left(l\right)}=\eta_{y}\left(W_{l\left(l-1\right)}^{V}\phi^{\prime }\left({\hat{y}}_{i}^{V\left(l-1\right)}\right)\left(y_{i}^{V\left(l-1\right)}-{\hat{y}}_{i}^{V\left(l-1\right)}\right)+\left(y_{i}^{V\left(l\right)}-{\hat{y}}_{i}^{V\left(l\right)}\right)\right)$$ (3)

where *η*_*y*_ is the rate for updating neuronal activities and *ϕ* ^*prime*^ is the derivative of the activation function used in the predictive coding network. The update rule in Eq. 3 is used to infer neuronal activity in all layers of the visual module for all inputs. Weights $\left(W_{l\left(l-1\right)}^{V}\right)$ between *lth* and (*l* − 1)_*th*_ layers in the network are updated by performing gradient descent on the error in the prediction generated by the *l*th layer neurons which results in the update rule for weights:

$$\Delta W_{l\left(l-1\right)}^{V}=\eta_{w}y_{i}^{V\left(l\right)}\phi^{\prime }\left({\hat{y}}_{i}^{V\left(l-1\right)}\right){\left(y_{i}^{V\left(l-1\right)}-{\hat{y}}_{i}^{V\left(l-1\right)}\right)}^{T}$$ (4)

where *η*_*w*_ is the learning rate for updating weights. Note that the learning rule is Hebbian-like in the sense that weight changes depend on the pre- and post-synaptic activity (pre: *y* ^*V*(*l*)^; post: $y^{V\left(l-1\right)}-{\hat{y}}^{V\left(l-1\right)}$ ). The learning approach for the tactile module is identical to the visual module. In case of the multisensory module, the representations are inferred based on prediction errors of topmost layers in both the visual and tactile modules.

### 4.2 Multi-Modal Variational Auto-Encoder Algorithm

Both JMVAE-zero and JMVAE-kl extend Variational Autoencoders (VAE) to handle multisensory inputs. Therefore, this section first provides a description of the VAE and then presents extensions pertaining to JMVAE-zero and JMVAE-kl.

#### 4.2.1 Variational Autoencoders

A VAE is an autoencoder with an encoder-decoder architecture that allows estimating a latent distribution which can be used to sample data from the input space. Given input data *x* with a distribution of *p*(*x*) and a prior distribution *p*(*z*), the encoder in a VAE estimates an approximate posterior distribution *q*_*ϕ*_(*z*|*x*) for the actual posterior *p* (*z*|*x*). Here, *ϕ* represents the parameters associated with the encoder. The decoder maximizes the likelihood of the data *p*_*θ*_(*x*|*z*) given this approximate posterior distribution where *θ* represents the parameters associated with the decoder. To overcome the intractable problem of computing the marginal distribution, VAEs are trained to maximize a lower bound for the input data distribution *p*(*x*) by maximizing the following objective function

$$L_{VAE}=-D_{KL}\left(q_{\phi }\left.\left(z|x\right)\|p\left(z\right)\right)\right)+E_{q_{\phi }\left(z|x\right)}\left(\log p_{\theta }\left(x|z\right)\right)$$ (5)

where the first term $D_{KL}\left(q_{\phi }\left(z|x\right)\|\left.p\left(z\right)\right)\right)$ represents the Kullback-Leibler divergence between the approximate posterior and the prior distribution *p*(*z*). The second term represents the reconstruction error in the output of the decoder. VAE’s represent both *q*_*ϕ*_(*z*|*x*) and *p*(*z*) using Gaussian distributions. The mean and variance of *q*_*ϕ*_(*z*|*x*) are determined by the output of the encoder. *p*(*z*) is assumed to be a standard normal distribution N(0,I) where *I* denotes the identity matrix. This assumption allows VAEs to estimate an approximate posterior that is closer to the standard normal distribution. This enables sampling from the learned latent distribution to generate samples from the input space.

**FIGURE 14:**
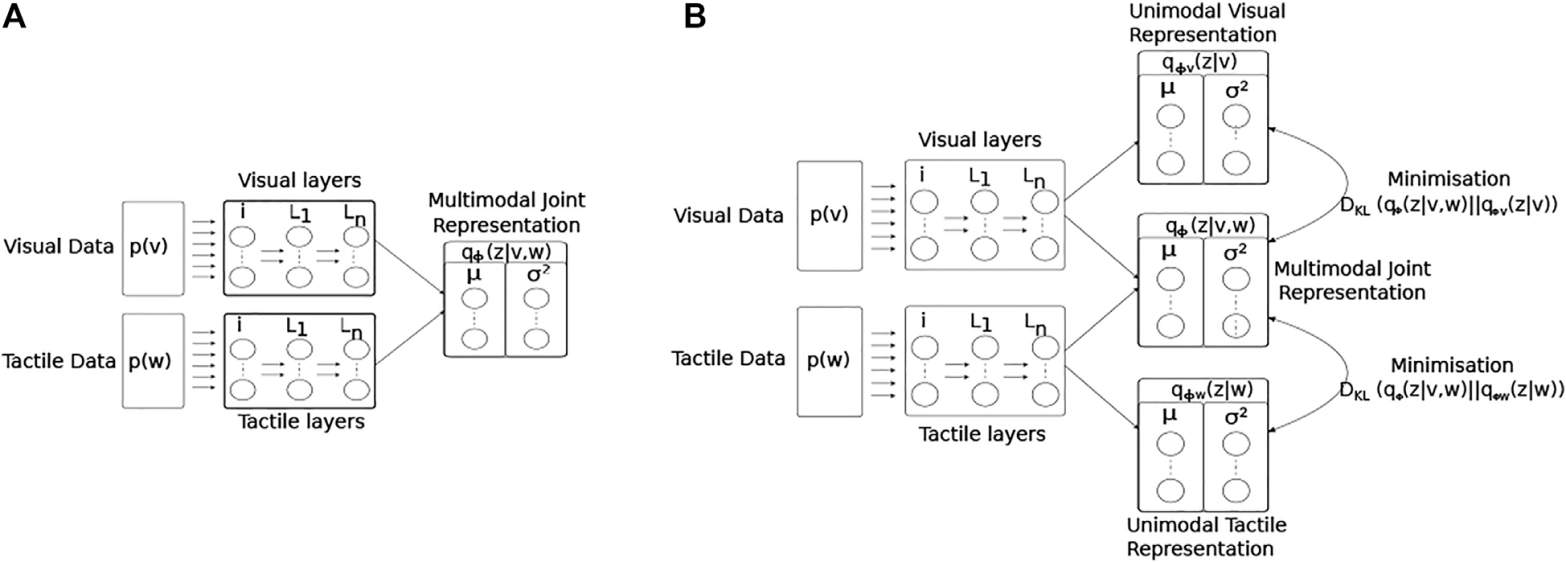
Architectural diagrams of the encoder networks of the two JMVAE models; **(A)** JMVAE-zero, and **(B)** JMAVAE-kl. Both networks attempt to represent the two input modalities (visual *p*(*v*) and tactile *p*(*w*)) as a joint multimodal latent representation *q*_*ϕ*_(*z*|*v*, *w*). This is done by minimising both the reconstruction error for each modality, and the KL-divergence (*D*_*KL*_) between a standard normal distribution and the joint multimodal distribution. The resulting continuous distribution is encoded by the activity of two parallel layers of nodes representing the mean and variance (*μ*, *σ* ^2^) of each latent dimension. The JMVAE-kl model trains 2 further encoders, one using only visual input *q*_*ϕv*_ (*z*|*v*) and the other only tactile *q*_*ϕw*_ (*z*|*w*), such that the KL-divergence measures between the unimodal and multimodal approximate distributions can be included into the loss function during training.

JMVAE builds upon VAE by enabling inference of joint representations based on input in multiple modalities. In this paper, multiple modalities constitute the input from tactile (denoted by *w* for whisker) and vision (denoted by *v*) sensors on the WhiskEye robot. A straightforward approach for inferring multimodal representations using a VAE is to learn a joint approximate posterior distribution *q*_*ϕ*_(*z*|*w*, *v*) using a network as shown in Figure 14A. In this approach, a VAE maximizes the following objective function to achieve a maximal lower bound on the marginal joint distribution

$$L_{JM}=-D_{KL}\left(q_{\phi }\left.\left(z|w,v\right)\|p\left(z\right)\right)\right)+E_{q_{\phi }\left(z|w,v\right)}\left(\log p_{\theta }\left(w|z\right)\right)+E_{q_{\phi }\left(z|w,v\right)}\left(\log p_{\theta }\left(v|z\right)\right)$$ (6)

Equation 6 represents the objective function for JMVAE-zero. It has been shown that JMVAE-zero is not able to generate good crossmodal reconstructions when there are large structural differences between different modalities Suzuki et al. (2017). To overcome this issue, a better VAE was developed in Suzuki et al. (2017) called JMVAE-kl. JMVAE-kl employs a JMVAE-zero with two additional encoders for inferring the approximate posteriors for the individual modalities *w* (denoted by *q*_*ϕw*_ (*z*|*w*)) and *v* (denoted by *q*_*ϕv*_ (*z*|*v*)) as shown in Figure 14B. It is trained using an objective function that minimizes the Kullback-Leibler divergence between the joint approximate posterior distribution and the approximate posterior distributions for individual modalities, given by

$$L_{JM\left(KL\right)}=L_{JM}-\alpha \left(D_{KL}\left(q_{\phi }\left(z|w,v\right)\|q_{\phi v}\left(z|v\right)\right)+D_{KL}\left(q_{\phi }\left(z|w,v\right)\|q_{\phi w}\left(z|w\right)\right)\right)$$ (7)

where *α* controls the strength of regularization due to the KL-divergence between the different posterior distributions, $L_{JM\left(KL\right)}$ encourages inference of similar multimodal and unimodal approximate posterior distributions thereby resulting in better crossmodal reconstructions.

### 4.3 RSA and Statistical Measures

#### 4.3.1 Representational Dissimilarity Matrix (RDM)

To transform the 100-dimensional representations inferred by each network model in response to each sample of a test set composed of *n* samples into an RDM, the dissimilarity distance between each representation to all others was calculated. In this case we use 1 - Pearson correlation coefficient as preferred by Kriegeskorte Kriegeskorte et al. (2008):

$$d_{x,y}=1-\frac{\sum \left(x_{i}-\bar{x}\right)\left(y_{i}-\bar{y}\right)}{\sqrt{\sum {\left(x_{i}-\bar{x}\right)}^{2}\sum {\left(y_{i}-\bar{y}\right)}^{2}}}$$ (8)

where *d*_*x*,*y*_ is the dissimilarity between representation x and y, which in turn will be the index address into the *n* x *n* symmetric RDM.

The RDMs for pose were constructed using the Euclidean distance between each 3D pose sample to all others in the test set. For correctness the orientation and position components of the poses (*x*_*rot*_ and *x*_*trans*_) were independently scaledBregier et al. (2018):

$$d_{x,y}=a\|x_{rot}-y_{rot}\|+b\|x_{trans}-y_{trans}\|$$ (9)

For the scaling factors *a* and *b* for rotation and translation respectively, we found that *a* = 0.3 and *b* = 1 to be appropriate in all experiments.

#### 4.3.2 Representational Similarity Analysis (RSA)

The vector of representational dissimilarity distances and accompanying vector of pose distances for each sample in the test set were sorted into rank order and compared using Spearman’s rank correlation coefficient (*ρ*):

$$\rho =1-\frac{6\sum \overset{2}{\underset{i}{d}}}{n\left(n^{2}-1\right)}$$ (10)

where *d*_*i*_ is the difference between the rank order in pose against representation distance, and *n* being the number of samples in the test set. The significance tests (*p*-values) were taken from $t=\rho \sqrt{\frac{n-2}{1-\rho^{2}}}$ which approximately follows Student’s t with *n* − 2 degrees of freedom under the null hypothesis.

#### 4.3.3 Precision-Recall Analysis

To build the ROC curve the True Positive Rate (TPR) and False Positive Rates (FPR) were calculated at each iteration of representation threshold to be tested as follows:

$$TPR=\frac{TP}{TP+FN}\;\;\;FPR=\frac{FP}{FP+TN}$$ (11)

Where the cumulative True Positive (TP), False Positive (FP), True Negative (TN) and False Negative (FN) scores from each iteration were used.

The geometric mean was calculated at each iteration as follows:

$$\sqrt{TPR*\left(1-FPR\right)}$$ (12)

## Data Availability

The raw data supporting the conclusions of this article will be made available by the authors, without undue reservation.
